# Differences between problematic internet and smartphone use and their psychological risk factors in boys and girls: a network analysis

**DOI:** 10.1186/s13034-023-00620-z

**Published:** 2023-06-12

**Authors:** Dmitri Rozgonjuk, Lukas Blinka, Nana Löchner, Anna Faltýnková, Daniela Husarova, Christian Montag

**Affiliations:** 1 Department of Molecular Psychology, Institute of Psychology and Education, Ulm University, Helmholtzstraße 8/1, 89081 Ulm, Germany; 2 Institute of Mathematics and Statistics, University of Tartu, Tartu, Estonia; 3 Psychology Research Institute, Faculty of Social Studies, Masaryk University, Joštova 10, 60200 Brno, Czech Republic; 4 Department of Health Psychology and Methodology Research, Faculty of Medicine, P.J. Safarik University in Kosice, Košice, Slovakia

**Keywords:** Problematic internet use, Problematic smartphone use, Psychology risk factors, HBSC, Network analysis

## Abstract

**Background:**

Problematic internet and smartphone use are significant health challenges for contemporary adolescents. However, their mutual relationship is unclear because studies investigating these phenomena are scarce. The present study aimed to investigate the psychological risks and protective factors associated with problematic internet and smartphone use.

**Method:**

A representative sample of Slovak adolescents (N = 4070, M_age_ = 14.38, SD_age_ = 0.77, 50.5% girls) from the Health Behavior in School-aged Children project was analyzed using network analysis separately for boys and girls.

**Results:**

The results showed weak (for boys) and moderate (for girls) associations between problematic internet use and problematic smartphone use. Risk factors showed stronger associations with problematic internet use than problematic smartphone use, with the exception of fear of missing out, which was strongly associated with problematic smartphone use. The central nodes were externalizing problems for boys and internalizing problems, externalizing problems, and resilience for girls.

**Conclusion:**

The study concluded that while problematic internet use and problematic smartphone use are somewhat related, they differ at the psychological level. In addition, the phenomena are rather different between boys and girls.

**Supplementary Information:**

The online version contains supplementary material available at 10.1186/s13034-023-00620-z.

## Introduction

Over the past two decades, increasing access to digital technology has transformed the lives of young people worldwide. Modern adolescents work with the internet and other digital technologies on an intensive daily basis [1]. The expansion and constant accessibility of internet and other technologies have created great opportunities for learning, work, entertainment, and personal exploration and growth [2]. However, intense online technology use may lead to various social and health risks, including reduced sleep quality [3], obesity [4], and reduced academic performance [5]. Concerns regarding excessive and potentially addictive use have been repeatedly expressed [6, 7]. Of the many different forms of excessive technology use, considerable attention has been paid to problematic internet use (PIU) and problematic smartphone use (PSU). Much research has already been devoted to these two phenomena in adolescence [8, 9], but the relationship between them (especially with regard to adolescents) has been studied to a limited extent. Our study fills this gap by comparing the similarities and differences between PIU and PSU in terms of their risk and protective factors (i.e., psychological variables that might affect susceptibility to PIU or PSU). Our focus is specifically on adolescent users, since they are often seen as a particularly vulnerable group in terms of the development of problematic forms of internet and smartphone use [1, 10].

Both PIU and PSU are operationalized as the inability to control one’s use of the medium, which leads to harmful consequences and disruptions in daily functioning [11, 12]. Even when facing these negative consequences, users have a diminished capacity to limit their time spent in the medium, and they are preoccupied with it, even when not online [13]. The term PIU covers a large number of excessive online activities, especially online gaming, social networking sites use (SNS), chatting, video watching, or online shopping [12]. Smartphones are internet-enabled devices providing instant and nearly unlimited access to online activities. In principle, when using a smartphone, the user is almost always simultaneously connected to the internet. Thus, in terms of user patterns, it can be assumed that the two phenomena overlap to a certain extent. On the other hand, the application use may be slightly different, because some social media use (e.g., Instagram, WhatsApp) is optimized for smartphones. The existing literature reports weak to strong correlations between PSU and PIU (e.g., r = 0.21 in Choi et al. [14]; r = 0.40 in Kwon et al. [15]; r = 0.50 in Lachmann et al. [16]; r = 0.64 in Škařupová et al. [17]). Although positive associations have been demonstrated, several studies have pointed out differences in usage patterns, gender, personality traits, and psychological variables between these two types of problematic behavior. While PSU has been found to be especially related (and almost identical) to social media use [18], extreme PIU scores were found to be related to online gaming [19]. In other words, both phenomena rely on somewhat different need satisfaction and anticipated rewards [20]. Specifically, in terms of internet usage, boys were reported to be more prone to addictive use than girls, whereas this pattern was reversed for smartphone use [14, 21]. Furthermore, lower extraversion was associated with higher PIU but was unrelated to PSU, whereas lower openness to experience was linked to higher PSU but not to PIU [22]. These results suggest that while PIU and PSU are related, a significant portion of unexplained variance remains that represents the differences between the constructs.

Both PIU and PSU are often studied in the context of users’ psychological characteristics and susceptibility to developing problematic forms of use. Previous meta-analyses [23–25] have suggested that the most consistent risk factors stem from the following areas: (1) high impulsivity and attention/hyperactivity disorders; (2) negative emotionality, anxiety, and depressive symptoms; and (3) low self-esteem and self-directedness. On the other hand, resilience and high self-control are often reported as the most important protective factors in terms of PIU and PSU [26, 27].

Impulsivity is frequently linked to addictive behaviors [28]. Internet users with higher impulsivity present executive dysfunction and deficient inhibitory control, which may contribute to problems with online technology use [29]. Together with disrupted self-control, attention problems, aggression, hyperactivity, and impulsivity are part of the construct of externalizing problems [30]. According to previous research, externalizing problems are relatively common in adolescent problematic media users and they were found to be significantly associated with PIU, specifically excessive social media use or internet gaming addiction [31–34].

In addition to externalizing problems, adolescents may also develop internalizing problems that include affective states, such as anxiety, social withdrawal, depression [35], diminished self-esteem, and feelings of hopelessness—all of which have been identified as risk factors in terms of developing both PIU and PSU [36–39]. It has been suggested that individuals with negative emotionality may tend to use smartphones or the internet excessively as a coping mechanism to eliminate distress [40, 41].

Internalizing problems in adolescence often go hand-in-hand with social anxiety, withdrawal from peer relationships, lack of social competence, and shyness [42, 43]. Thus, these individuals experience problems with social functioning and social inclusion [44]. Adolescents who need to belong and whose social connections are unsatisfied in real life might tend to fulfill these needs through SNS [45]. Przybylski et al. proposed that fear of missing out (FoMO) may explain these dynamics. Fear of missing out is defined as a pervasive apprehension that others might have more rewarding experiences or acquire useful information that one does not have access to [46]. To not miss something important on the site, people with increased FoMO feel the need to be as often online as possible. They often experience anxiety offline and feel pressured to constantly check for new information [47]. Previous research has demonstrated robust associations between FoMO and problematic social networks use [48], PIU [49], and PSU [50]. A potential explanation to these findings may lie in unmet social needs: it has been demonstrated that adolescents with higher FoMO also tend to have a higher need for popularity/belonging as well as higher social media use intensity [45].

Resilience is one of the strongest protective factors for PIU or PSU. Resilience is a multidimensional construct defined as the ability to adapt positively to life conditions and thrive, even in the face of adversity [51]. A resilient individual can use constructive coping strategies to successfully deal with adverse life events [52]. Resilience has been repeatedly suggested as a protective factor against various psychopathologies and risky behaviors, including internet, smartphone, and social media addiction [53–56]. Young people face many stressful challenges due to biological, psychological, and social changes. Adolescents with higher resilience have better internal resources to cope with stressful events, which might lead them to become less involved in using the internet to regulate negative emotions [57].

Although a relatively large body of work has examined PIU and PSU use separately, examinations of both are relatively scarce. Based on the literature review, we assume that they are related (e.g., share some of the predictors), but distinct phenomena. Owing to the different usage patterns of boys and girls, we further assumed that the differences would be reflected at the gender level as well. Thus, the aim of this study was to examine what psychological risk and protective factors are shared by PIU and PSU and what factors define the dividing line. With the help of a network analysis, we aim to examine the association separately for boys and girls.

## Methods

### Data collection and sample

In the present study, we used data from the WHO-collaborative Health Behavior in School-aged Children (HBSC) study [58]. The HBSC is a cross-sectional study carried out at 4-year intervals in 50 countries and regions across Europe and North America. Only the survey conducted in Slovakia in 2018 was analyzed because it used the key variables essential for the purpose of this study. A nationally representative sample of Slovak adolescents aged 11–15 was obtained using a two-step data collection procedure. First, the list of all eligible schools in Slovakia was obtained from the Slovak Institute of Information and Prognosis for Education, and then stratified by region and type of school (primary vs. secondary school). A total of 140 schools were randomly selected and asked to participate; and 109 agreed to participate (response rate 77.85%). In the second step, one class from each grade within the target age group was randomly selected from each school. Data were collected anonymously using self-report electronic questionnaires administered by trained administrators during classroom sessions. Participation was voluntary, and passive parental informed consent was obtained before administering the questionnaires.

The sample included more than 8405 Slovakian adolescents aged 11–15. However, some of the key variables (including the PSU scale) were administered only to respondents aged 13–15, which reduced the sample size to 5053 participants. Moreover, we removed the data of students whose responses were missing for at least 75% of the scale items used in this study. Therefore, the effective sample, which comprised 4070 adolescents (age M = 14.38, SD = 0.77; 2013 boys and 2057 girls), was used in this study. Participants' gender was not associated with missing data. The average age of the excluded samples (M = 14.24) was lower than that of students who were not excluded from the dataset because of missing data (M = 14.38), t(1384.6) = − 4.915, *p* < 0.001. Data for other study participants who had missing data were imputed (see the analysis section for details).

### Measures

In the current study, we used data that included participants' sociodemographic characteristics and their responses to scales that assessed the severity of PIU and PSU. They also included experiencing externalizing and internalizing problems, fear of missing out, resilience, and hopelessness. Descriptive statistics for these scales and their internal consistencies are presented in Table 1.

**Table 1:** Descriptive statistics of the total sample including boys and girls

Variable	Theoretical range	Total sample (N = 4070)			Boys (N = 2013)		Girls (N = 2057)		Boys–girls difference test		
		M	SD	α	M	SD	M	SD	*W*	*p*	*d* (95% CI)
PIU	(5, 20)	8.00	3.02	0.79	7.94	3.20	8.06	2.84	1,928,820	< 0.001	0.04 (− 0.02; 0.10)
PSU	(9, 45)	23.36	8.24	0.86	22.36	8.61	24.33	7.74	1,750,946	< 0.001	0.24 (0.18; 0.30)
Externalizing	(10, 30)	16.67	3.27	0.66	16.53	3.22	16.81	3.31	1,981,714	0.018	0.09 (0.02; 0.15)
Internalizing	(10, 30)	15.38	3.35	0.63	14.63	3.13	16.12	3.40	1,523,921	< 0.001	0.45 (0.39; 0.52)
FoMO	(5, 25)	12.68	4.01	0.73	11.99	4.21	13.36	3.68	1,623,190	< 0.001	0.35 (0.29; 0.41)
Resilience	(12, 36)	29.29	3.68	0.68	29.27	3.59	29.30	3.77	2,042,408	0.434	0.01 (− 0.05; 0.07)
Hopelessness	(5, 10)	5.85	1.38	0.79	5.75	1.27	5.95	1.47	1,965,174	0.001	0.14 (0.08; 0.20)

PIU, problematic internet use; PSU, problematic smartphone use; FoMO, fear of missing out; W, Wilcoxon rank sum test statistic; d, Cohen's d group differences effect size statistic

*PIU* was measured using the Excessive Internet Use Scale [EIU; 59]. The scale consists of five items covering five of the six factors of the Griffiths component model of behavioral addiction [60]. These factors are salience (i.e., "I have gone without eating and sleeping because of the internet"), withdrawal symptoms (i.e., "I have felt bothered when I cannot be on the internet"), tolerance (i.e., "I have caught myself surfing when I am not really interested"), relapse (i.e., "I have tried unsuccessfully to spend less time on the internet"), and conflict (i.e., "I have spent less time than I should with either family, friends, or doing schoolwork because of the time I spend on the internet"). Participants used a 4-point scale (ranging from 1 = "never" to 4 = "very often") to express how often they had experienced certain symptoms in the preceding 12 months. The final variable was the sum of the five items.

*PSU* was measured using the Problematic Mobile Phone Use Scale [MPPUS-10; 61]. This is a shortened version of the Mobile Phone Problem Use Scale [62] and consists of 10 items (e.g., "I have used my mobile phone to make myself feel better when I was feeling down"). Participants answered on a 5-point scale (1 = "strongly disagree", 5 = "strongly agree") to what extent they agreed with each statement about their everyday mobile use. A higher score indicated more severe symptoms of PSU. Of note, because the study was conducted in adolescents of whom most were not financially independent of their parents/caretakers, the last item of MPPUS-10 (“I have received mobile phone bills I could not afford to pay”) was not included in the analyses. Hence, the final variable was created as the sum of nine items.

*Internalizing problems and externalizing problems* were assessed using the Strengths and Difficulties Questionnaire [SDQ; 63]. The original questionnaire consisted of 25 items, but the prosocial behavior subscale was omitted from the HBSC, so the scale consisted of 20 items that covered four subscales: emotional symptoms (i.e., "I am often unhappy, downhearted or tearful"), conduct problems (i.e., "I get very angry and often lose my temper"), hyperactivity (i.e., "I am restless; I cannot stay still for long "), and peer relationship problems (i.e., "Other children or young people pick on me or bully me"). Five items were reverse coded and rescaled for further analysis. Each item had three answer options: 1 = "not true", 2 = "somewhat true", and 3 = "certainly true", with a higher score indicating more internalizing of problems or externalizing of problems. Instead of four individual subscales we decided to work with broader internalizing and externalizing problem subscales because they were shown to work better in generalized (nonclinical) populations [64]. Therefore, we combined emotional and peer subscales into an internalizing problem subscale, and behavioral and hyperactivity subscales into an externalizing problem subscale. Both variables were calculated as the sum of the scores for each item.

*Fear of missing out* was measured using a shortened version of the Fear of Missing Out Scale [FoMO; 46]. Unlike the original 10-item questionnaire, we used a five-item version for each statement, ranging from 1 = "strongly disagree" to 5 = "strongly agree.” The final variable was computed as the sum of the scores for the five items.

*Hopelessness* was measured using the Hopelessness Scale for Children [HSC; 65], which is a five-item tool with answer categories 1 = “agree” and 2 = “disagree” (e.g., "All I see ahead of me are bad things, not good things"). The sum of the scores for the five items was computed.

*Resilience* was measured using the shortened version of the Child and Youth Resilience Measure [CYRM-12; 66]. This measure is based on the socio-ecological definition of resilience, which implies that individual, peer, family, school, and community resources contribute to positive outcomes for youth. The scale consists of 12 items that cover all previous factors (e.g., "Do you have chances to show others that you are growing up and can do things by yourself?"). For each item, participants chose between three options: 1 = "no"; 2 = "sometimes"; and 3 = "yes". The sum of the scores of the 12 items was calculated.

### Analysis

Data were analyzed using the R software v4.1.3 [67]. First, we analyzed the missing data. As mentioned previously, the participants who did not respond to at least 75% of the items on each scale were excluded from the analyses. For the rest of the sample, if there was missing data, the data were imputed using predictive mean matching with the mice package v3.14.0 [68]. Internal consistency statistics, Fisher's r-to-z transformation, which is based on correlation-difference testing (for correlations that included either PIU or PSU), and Cohen's d-s were computed using the functions in the *psych* package v2.2.3 [69]. Pearson’s correlation coefficients were computed as statistics for the associations between variables of interest. The Wilcoxon rank-sum test was used to compute the mean differences between boys and girls.

To evaluate the complexity of the associations between psychological variables and PIU and PSU, we estimated two Gaussian graphical models [GGM; 70] with the summed scores for PSU and PIU, and other psychological variables. The plot of networks includes edges and nodes; the former depicts association strength and direction (positive or negative), whereas the latter marks the variables. According to Rodebaugh et al. [71], the strongest nodes are those that have the most relationships with other variables in the networks such that a change in those central nodes would have a significant impact on changes in all other variables. Researchers have previously suggested that the strength of a node is a crucial index for identifying variables for developing the most effective interventions [72]. The edges in GGM are conditionally dependent relationships between the nodes. The graphical least-absolute shrinkage and selection operator, in combination with the Extended Bayesian Information Criterion (EBICglasso) model selection was used to estimate GGM [73] for parsimonious/sparse networks. In addition, all nodes were predicted by other nodes for node predictability statistics. To assess the accuracy of the network centrality estimates, case-drop bootstrapping (over 1000 permutations) was computed, and bootstrapped difference tests were run to test the differences in edge weights and node centrality. Finally, we computed the centrality stability (CS) coefficients for both models; a large coefficient indicates that the estimated centrality measure is robust [74]. The packages *qgraph* v1.9.2 [75], *bootnet* v1.5 [74], and *mgm* v1.2.12 [76] were used for network analysis.

## Results

### Descriptive statistics and correlation analysis

Descriptive statistics and correlation analysis results are presented in Tables 1 and 2, respectively. The correlations for boys and girls are shown separately in Additional file 4: Table S1.

**Table 2:** Correlation analysis results for the total sample

Variable	Total sample (N = 4070)							
	1	2	Paired correlation difference test (PIU-PSU)		3	4	5	6
1. PIU	–							
2. PSU	0.298***	–	***T***	***p***				
3. Externalizing	0.344***	0.230***	6.541	< 0.001	–			
4. Internalizing	0.275***	0.200***	4.208	< 0.001	0.416***	–		
5. FoMO	0.206***	0.308***	− 5.775	< 0.001	0.247***	0.238***	–	
6. Resilience	− 0.244***	− 0.076***	− 9.298	< 0.001	− 0.405***	− 0.405***	− 0.094***	–
7. Hopelessness	0.262***	0.149***	6.292	< 0.001	0.321***	0.423***	0.176***	− 0.426***

PIU, problematic internet use; PSU, problematic smartphone use; FoMO, fear of missing out

***p < 0.001

Differences between boys and girls in the mean values of all of the variables were tested using the Wilcoxon rank sum test. As shown in Table 1, girls scored significantly higher on all measured variables, except for resilience, where the difference between boys and girls was not significant. The results in Table 2 show that PIU and PSU are moderately positively correlated. Furthermore, both PIU and PSU were positively correlated with both externalizing and internalizing symptoms, FoMO and hopelessness scores with small-to-moderate effect sizes. A small negative correlation was also observed between resilience and both PSU and PIU.

Differences in correlations between PIU and PSU and other variables were tested using Fisher's r-to-z transformation. Because the correlations were tested using the dependent sample, paired correlation-difference testing was used. In the total sample, PIU showed significantly stronger positive correlations with both externalizing and internalizing symptoms and hopelessness and a stronger but negative correlation with resilience, while PSU showed a stronger correlation with FoMO. These results are consistent with the values for the separate samples of girls and boys.

Table 3 presents the results of the unpaired correlation-difference tests between boys and girls in PIU and PSU associations with other variables. In the sample of boys, the correlation between PIU and PSU was weak. In the sample of girls these two variables correlate moderately strongly, and the difference between correlation values is significant. In the case of PIU, we observed gender differences with regards to FoMO; for girls, the correlation between PIU and FoMO was significantly stronger than that for boys. In PSU, its correlation with other variables is, in all cases, significantly stronger in the sample of girls, except for FoMO.

**Table 3:** Correlation differences between boys and girls in associations including PIU and PSU

Variable	PIU				PSU			
	Boys	Girls	z	p	Boys	Girls	z	p
PIU	–	–	–	–	0.190	0.430	8.528	** < 0.001**
PSU	0.190	0.430	8.528	** < 0.001**	–	–	–	–
Externalizing	0.328	0.361	1.194	0.233	0.172	0.287	3.875	** < 0.001**
Internalizing	0.252	0.306	1.867	0.062	0.148	0.212	2.109	**0.035**
FoMO	0.161	0.261	3.339	**0.001**	0.270	0.325	1.924	0.054
Resilience	− 0.250	− 0.240	0.339	0.735	− 0.040	− 0.115	2.406	**0.016**
Hopelessness	0.248	0.277	0.993	0.321	0.106	0.178	2.343	**0.019**

PIU, problematic internet use; PSU, problematic smartphone use; FoMO, fear of missing out. Statistically significant correlation differences are highlighted in bold font

### Network analysis

**Fig. 1:**
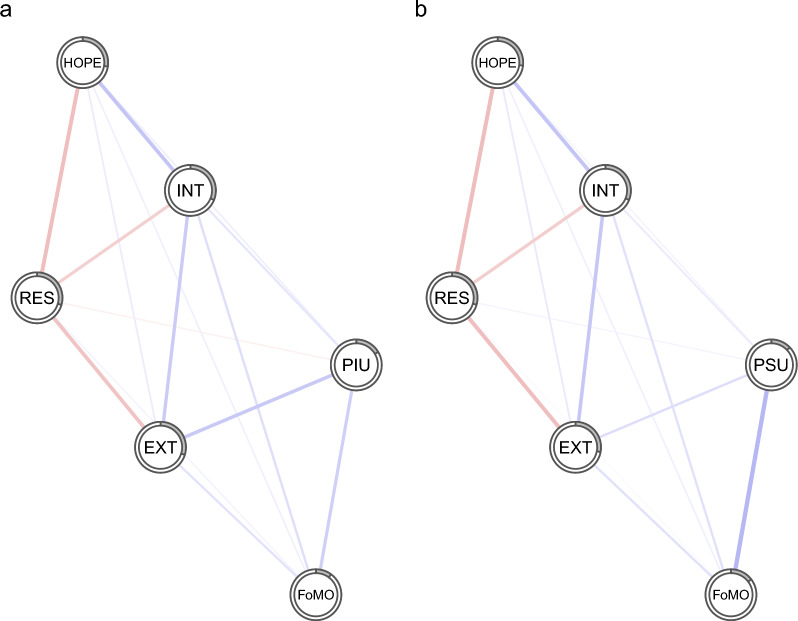
Regularized partial correlation networks that include either PIU (**a**) or PSU (**b**) in association with other psychological variables in the total sample. *Notes*: Blue lines represent positive regularized partial correlations and red lines represent negative regularized partial correlations. The line thickness indicates the strength of the relationship. The grey pie chart that surrounds each node depicts the proportion of a given node's variance, as explained by the other nodes in the network. PIU, problematic internet use; PSU, Problematic smartphone use; FoMO, fear of missing out; INT, Internalizing; EXT, Externalizing; HOPE, Hopelessness; RES, Resilience

**Fig. 2:**
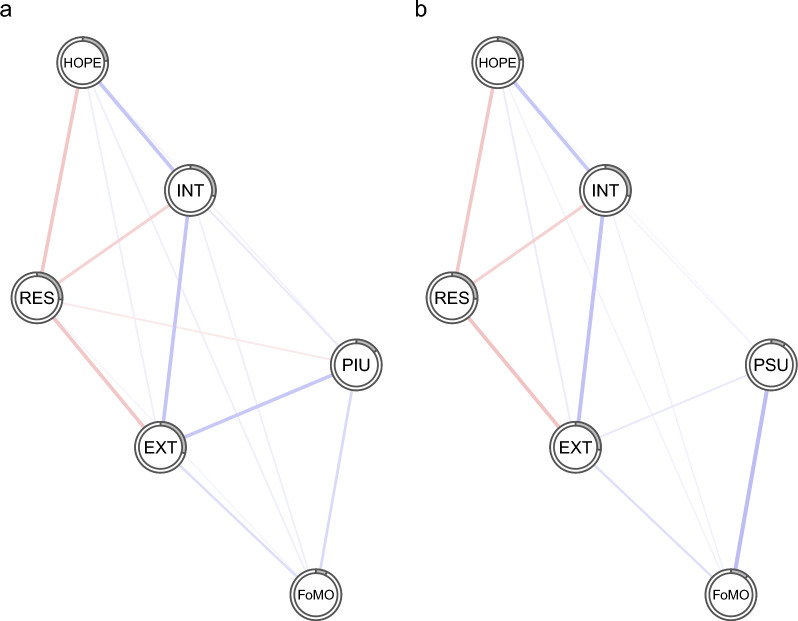
Regularized partial correlation networks that include either PIU (**a**) or PSU (**b**) in association with other psychological variables in the boys' sample. Notes: Blue lines represent positive regularized partial correlations and red lines represent negative regularized partial correlations. The line thickness indicates the strength of the relationship. The grey pie chart surrounding each node depicts the proportion of a given node's variance, as explained by the other nodes in the network. PIU, problematic internet use; PSU, Problematic smartphone use; FoMO, fear of missing out; INT, Internalizing; EXT, Externalizing; HOPE, Hopelessness; RES, Resilience

**Fig. 3:**
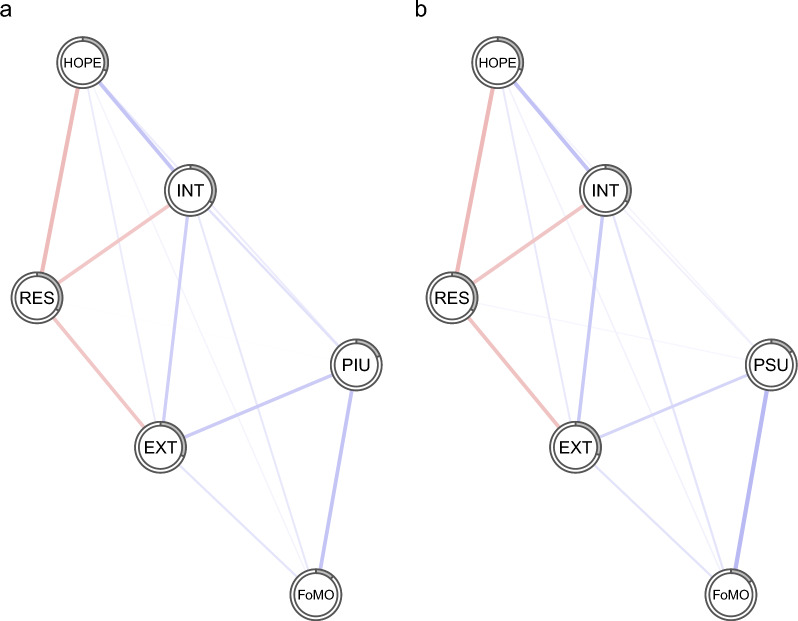
Regularized partial correlation networks that include either PIU (**a**) or PSU (**b**) in association with other psychological variables in the girls' sample. Notes: Blue lines represent positive regularized partial correlations and red lines represent negative regularized partial correlations. The line thickness indicates the strength of this relationship. The grey pie chart surrounding each node depicts the proportion of a given node's variance, as explained by the other nodes in the network. PIU, problematic internet use; PSU, Problematic smartphone use; FoMO, fear of missing out; INT, Internalizing; EXT, Externalizing; HOPE, Hopelessness; RES, Resilience

To investigate the associations between PIU, PSU, and other psychological variables in a more complex framework, we computed two regularized partial correlation networks, which involved PIU and PSU, for the total sample and for boys and girls separately, to determine whether these specific variables were differentially associated with psychological variables. These networks are depicted in Figs. 1, 2 and 3. The edge weights of the edges of the networks in Figs. 1, 2 and 3 are in Additional file 5: Table S2.

Figures 1, 2 and 3 show that, in a large part, the networks appear very similar. One distinction between boys’ and girls’ networks is that in boys, resilience has a small negative association with PIU, whereas in girls, there is a slight positive association between resilience and PSU. The average node predictability for networks, including PIU and PSU, was R^2^ = 0.241 and R^2^ = 0.238, respectively. The average node predictability statistics for the boys' and girls' networks were roughly of similar magnitude, with R^2^_PIU_ = 0.216 and R^2^_PSU_ = 0.208 for boys, and R^2^_PIU_ = 0.265 and R^2^_PSU_ = 0.262 for girls.

**Fig. 4:**
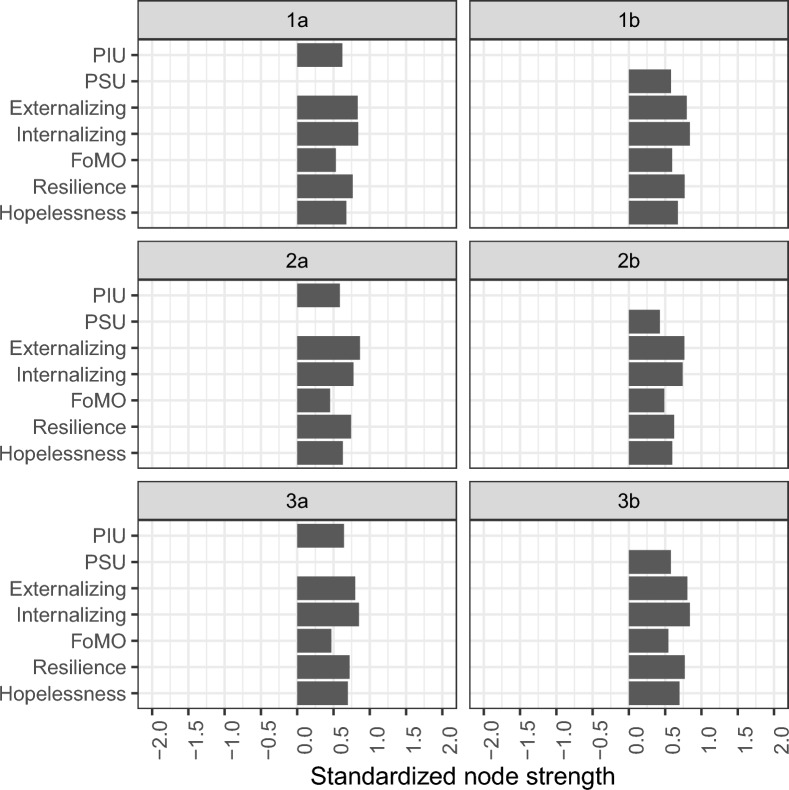
Node strengths for the networks depicted in Fig. 1, 2 and 3. PIU, problematic internet use; PSU, Problematic smartphone use; FOMO, fear of missing out; INT, Internalizing; EXT, Externalizing; HOPELESS, Hopelessness

In all cases, the networks showed acceptable stability, with a centrality stability coefficient of CS ≥ 0.70. The node strengths of these models are shown in Fig. 4. Figure 4 shows the PSU and PIU cannot be characterized as central nodes in any network. Potential differences in node centralities were also observed. Specifically, in the boys' sample (2a and 2b tabs in Fig. 4), the most central nodes were for externalizing symptoms and resilience, while, among girls, the most central nodes seemed to be both the externalizing and internalizing symptoms.

Additional network statistics (edge weights and node strength difference test results) are presented in Additional files 1, 2 and 3. In general, it could be observed that most of the node strengths were statistically significantly different from each other. However, in all models, the externalizing and internalizing factor nodes had the highest node strength values, and these factors were not statistically different from each other. With regard to the edge difference test results, most of the edges were statistically significantly different from each other. However, in networks involving PIU, the edges PIU-hopelessness and PIU-externalizing factors were not statistically different from each other.

## Discussion

The current study aimed to investigate the associations between adolescents’ PIU, PSU, and related protective and risk factors. Specifically, we examined the extent to which PIU and PSU share the same risk and protective factors, and how these variables are interrelated in boys and girls. The findings showed that PIU and PSU were positively correlated; however, this association was weak in boys and moderate in girls. PIU and PSU showed a roughly similar structure of relationships with other variables—they were both positively associated with psychological risk factors. However, the correlations between PIU and the other variables were significantly stronger than those between the same variables and PSU, with FoMO as the only exception. The relationships studied also differed between boys and girls.

There can be several reasons why the associations between PIU and psychological variables were stronger than those between PSU and psychological factors. One potential reason is that children may have more access to internet-based activities via devices other than smartphones (e.g., PC, tablets, etc.). This may result in more uninterrupted time spent online, as it may be plausible that smartphones prompt interruptive notifications more frequently than, say, tablets. Owing to fewer interruptions, children may have extended their screen time with an activity. It should also be noted that when it comes to assessing internet use, the line between the use of online functionalities of a smartphone may be implicitly included in the evaluation, as PIU may be an umbrella concept covering other online-based problematic behaviors [77, 78]. Another potential explanation could be that internet use may lead to a sense of anonymity when not performed on a smartphone. In other words, one could hypothesize that a smartphone may be associated with reduced online disinhibition [79], because communication with disclosed contacts may create a feeling of lower anonymity. Online anonymity, in turn, may promote lurking behavior—socially passive internet consumption—which has been shown to be associated with reduced mental health and problematic social media use [80, 81].

Based on these results, we cannot claim that the PIU and PSU exhibit the same phenomenon. Their mutual correlation was relatively low and they shared approximately 8.8% of the variance in the total sample. In contrast, our study showed that PIU and PSU had very similar relationships with the psychological variables. They both showed a positive association with fear of missing out, hopelessness, externalizing problems, and internalizing problems, and a negative association with resilience. These variables clearly contributed to the shared variance between PIU and PSU. This is consistent with previous studies that showed that fear of missing out, externalizing problems (i.e., impulsivity, hyperactivity, aggression), and internalizing problems (i.e., various emotional difficulties) could be risk factors for various forms of problematic online behavior [34, 36, 49], whereas resilience is a protective factor in these cases [55, 56]. However, it must be noted that the associations between selected variables and PIU were stronger than their associations with PSU, which is in line with the study by Jeong et al. [82], who found that the risk factors for PIU were different from those of PSU and non-addicted groups. These results raise questions regarding the extent to which PSU is an independent pathological phenomenon. The only variable, whose association with PSU was stronger than with PIU, was the fear of missing out. It was previously found that people who scored high on FoMO had a higher tendency to overuse their smartphones to satisfy their need for constant connectedness [83]. Owing to their portability, smartphones can provide 24/7 internet access allowing users to constantly check what is happening online. At the same time, this permanent connectedness heightens the awareness of possibly missing out on potentially more rewarding experiences, which could fuel FoMO even more [84].

Our findings also indicate interesting differences between girls and boys. With the exception of resilience, girls showed significantly higher values for all measured variables than boys. There are several possible reasons why the associations between PIU, PSU, and psychological variables are generally stronger in girls than boys. It should be noted that similar results have been demonstrated before; specifically, girls tend to spend more time online (and on digital devices) than boys, and problematic digital technology use has also been reported higher in girls than boys [85]. Given that girls place greater importance on social relationships and it also affects their mental well-being more than in boys [86] it is also natural that, for instance, social media usage patterns differ across genders [87]. It has been demonstrated that girls are more affected by online social comparisons [88], which could affect their body image [89]. This could, subsequently, also affect other aspects of mental health.

As for PSU, however, current research states that gender differences are not as evident, although some authors have reported that females are more susceptible to PSU [90, 91]. Recently, it has been found that girls use SNS and other social communication channels much more intensively, such as Facebook Messenger and WhatsApp [92, 93]. These applications are available predominantly on smartphones, which could partially explain why girls exhibit higher PSU values than boys do. In the case of PIU, the latest research predominantly states that boys have higher levels of PIU than girls [e.g., 94, 95], while others are in line with our findings [e.g., 96–98]. For example, Ha and Hwang [96] found that girls with emotional difficulties had a higher risk of developing internet addiction than boys with the same conditions. As the girls in our sample reported higher levels of emotional problems than boys, it is possible that these factors may have been related to more PIU. Girls also scored significantly higher on all psychological variables, except resilience, for which no difference was found between boys and girls. This result is consistent with previous studies that repeatedly report a higher prevalence for mental health problems in girls compared to boys [99–101]. During adolescence, girls are more susceptible to specific stressors associated with increased psychological distress and an increased likelihood of mental health problems, such as body dissatisfaction [102], low self-esteem [99], and academic stress and worries about school performance [103]. According to some authors [104, 105] boys may have more difficulties acknowledging and describing their mental health issues and, in comparison to girls, tend to mask or downplay their problems. This may be related to cultural expectations related to gender roles—in many societies, boys are discouraged from showing vulnerability or weakness and, thus, tend to complain less often about their health problems in general [106].

The results of the network analysis also showed that PIU and PSU were not the central nodes in any network. For boys, the node with the highest strength was externalizing problems, whereas for girls, resilience, externalizing problems, and internalizing problems were of comparable importance. In the present study, this could mean that, if the central nodes (e.g., resilience, externalizing problems, internalizing problems) are targeted, they can significantly change the levels of PIU and PSU; thus, they are ideal targets for prevention and treatment. The importance of internalizing and externalizing problems as risk factors for the development of PIU or PSU has been demonstrated previously. For example, internalizing problems, depression, anxiety, and peer-relationship difficulties predicted both PIU and PSU in previous studies [36, 37, 56, 107]. In the case of externalizing problems, impulsivity, aggression, and attention deficit hyperactivity symptoms have been previously identified as risk factors for PIU or PSU [56, 107–110]. Some studies [101, 111] also suggest that boys have a higher tendency to externalize problems and girls internalize problems, which could explain why internalizing problems are the central node in girls’ networks but not in boys’ networks. At the same time, resilience has been reported to be one of the most important protective factors against the development of PIU or PSU [54, 55].

The results of our study can be used to develop intervention programs to prevent PIU and PSU. As mentioned above, targeting central nodes in a network may lead to improved mental health. As an example, externalizing symptoms were among the nodes with the highest strength in both boys and girls, meaning indicating that targeting these symptoms may lead to improved well-being in children. There are several examples of how externalizing symptoms can be addressed in children. For instance, parent training programs could be useful in teaching how to cope with children’s behavior by communicating clear expectations, providing consistent consequences for misbehavior, and reinforcing positive behaviors [112]. Social skills training may also have beneficial effects on externalizing symptoms [113]. Finally, school-level interventions (e.g., mental health literacy and stigma mitigation) could also be useful in reducing the severity of externalizing symptoms [114].

This study has several limitations. First, because the design was cross-sectional, causal relationships between variables could not be inferred based on the results. Second, the study design only entailed an interindividual perspective. Previous research on individual differences, however, demonstrated that the structure and associations of inter- and intraindividual differences might not necessarily be the same [e.g., 115]; thus, future studies should incorporate an intraindividual perspective next to the interindividual perspective [116]. Third, this study did not include other factors, such as family, in the present analyses. It has previously been demonstrated that children’s family circumstances, such as parental education [117], might be associated with children’s digital device use [118]. Future research should consider intraindividual differences in PIU and PSU within this context. Fourth, the study used self-reported data, which might be prone to response bias such as social desirability or acquiescence. Fifth, since the data came from a complex epidemiological study, it was not always possible to use full-length questionnaires; therefore, shortened versions of most scales were used instead. This could have negatively affected the reliability of the scales. At the same time, both of the PIU and PSU scales are designed to measure generalized internet and smartphone addiction; therefore, they cannot provide information about specific types of internet or smartphone usage behaviors (e.g., social networking, gaming, and online shopping). Despite these limitations, the key advantage of our study was its large and nationally representative sample of adolescents.

## Conclusion

PIU and PSU are weakly to moderately related phenomena, yet they are distinct constructs that differ at the psychological level, with psychological risk factors mostly being especially relevant for PIU. Moreover, these phenomena were rather different between boys and girls, with stronger associations between PIU and PSU and psychological risk factors in girls.

## Supplementary Information

**Additional file 1.** Supplementary Figure S1: Bootstrapped edge weigths (95% CIs)

**Additional file 2.** Supplementary Figure S2: Edge weights differences test results

**Additional file 3.** Supplementary Figure S3: Centrality difference test results

**Additional file 4.** Supplementary Table S1: Correlation analysis results for the total sample, boys, and girls

**Additional file 5.** Supplementary Table S2: Edges

## Data Availability

The dataset used and analyzed during the current study are available from the corresponding author Lukas Blinka on scholarly request.

## Abbreviations

CS: Centrality stability
CYRM-12: Child and Youth Resilience Measure
EIU: Excessive Internet Use Scale
EBICglasso: Extended Bayesian Information Criterion
FoMO: Fear of missing out
GGM: Gaussian graphical models
HBSC: WHO-collaborative Health Behavior in School-aged Children study
HSC: Hopelessness Scale for Children
MPPUS-10: Problematic Mobile Phone Use Scale
PIU: Problematic internet use
PSU: Problematic smartphone use
SDQ: Strengths and Difficulties Questionnaire
SNS: Social networking sites
