# Supplementary figures and images for: Differences between problematic internet and smartphone use and their psychological risk factors in boys and girls: a network analysis
Source: Child Adolesc Psychiatry Ment Health. 2023 Jun 12;17:69. doi: 10.1186/s13034-023-00620-z (PMC10262453; doi:10.1186/s13034-023-00620-z)

Supplementary Figure S1.  
Bootstrapped edge weights (95% CIs)

1a (PIU)

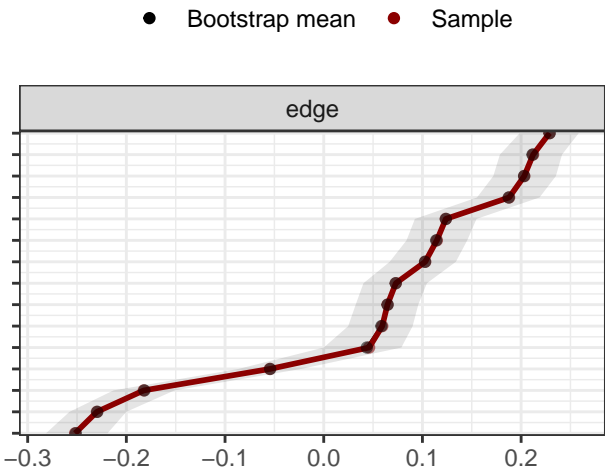

1a (PSU)

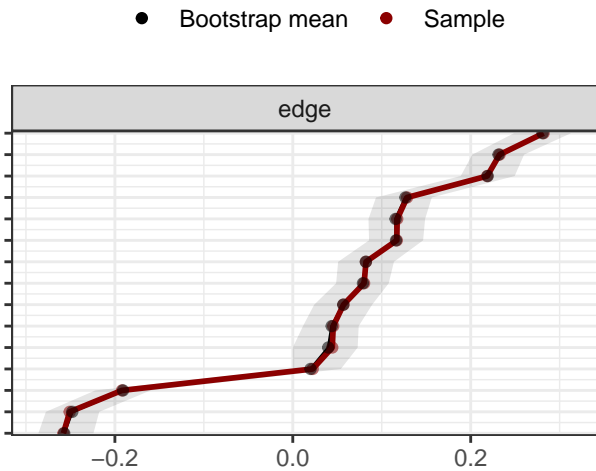

2a (PIU)

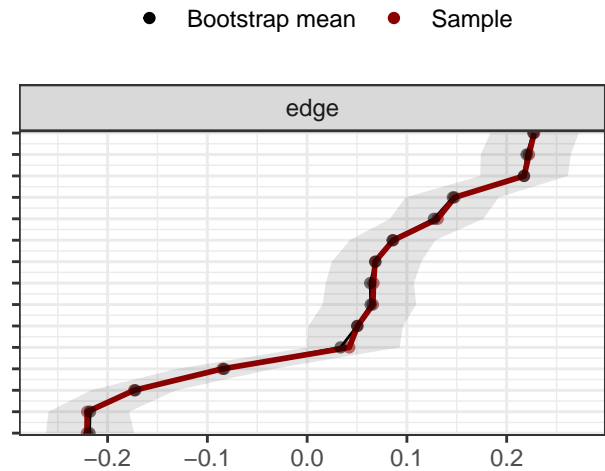

2a (PSU)

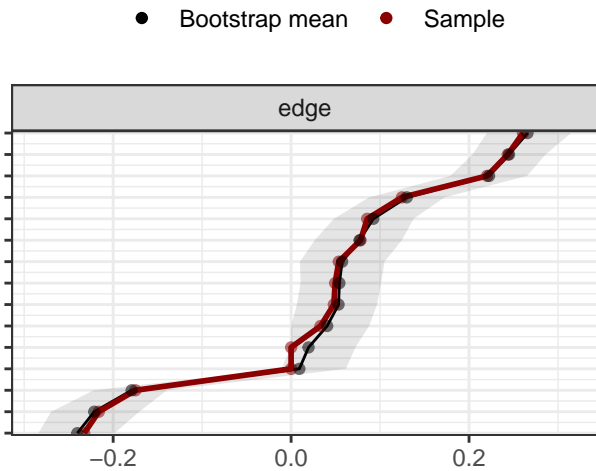

3a (PIU)

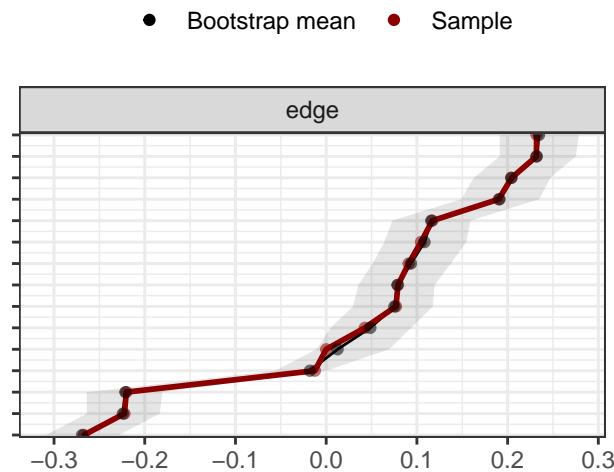

3a (PSU)

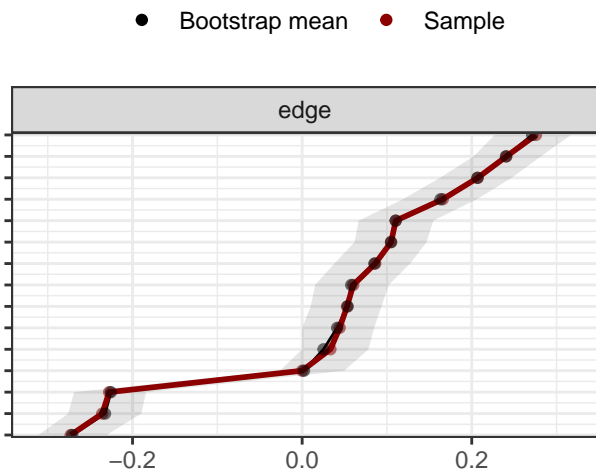

Supplement: Supplementary file 1 — Additional file 1. Supplementary Figure S1: Bootstrapped edge weigths (95% CIs) [file 13034_2023_620_MOESM1_ESM.pdf]

Supplementary Figure S2.  
Edge weights differences test results

1a (PIU)

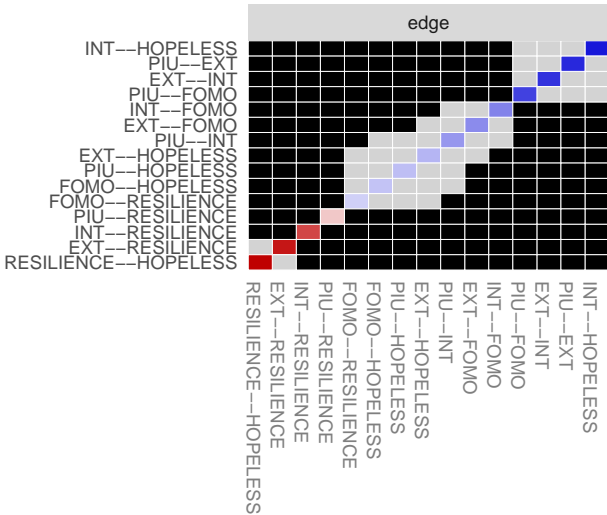

1a (PSU)

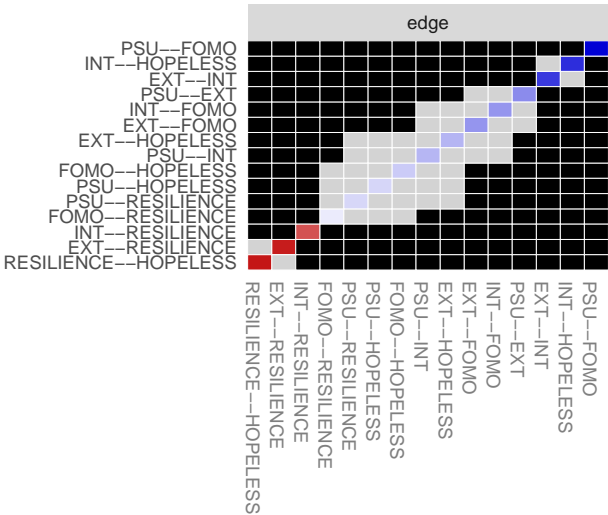

2a (PIU)

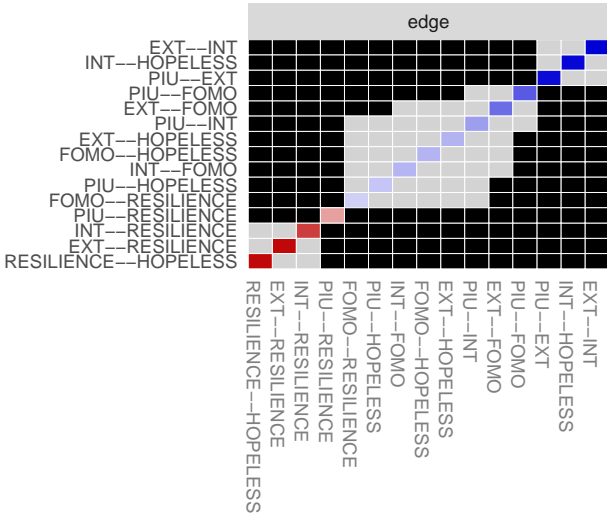

2a (PSU)

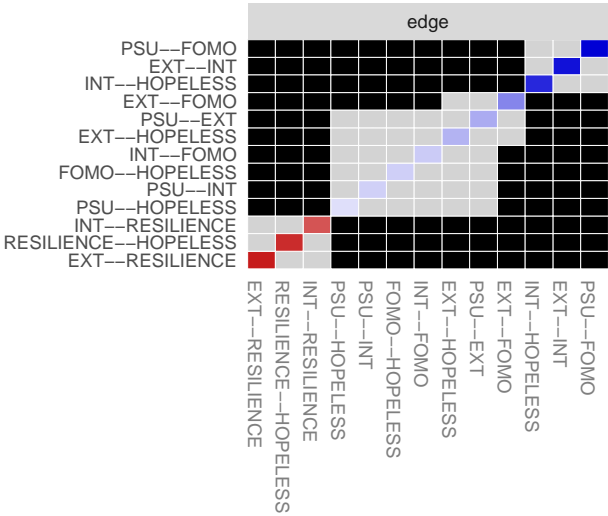

3a (PIU)

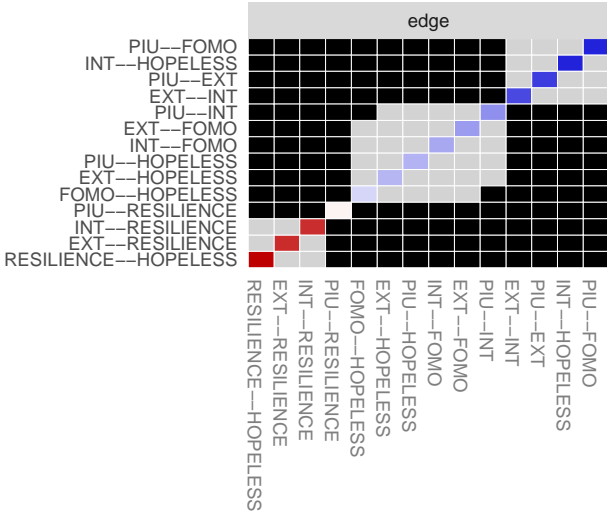

3a (PSU)

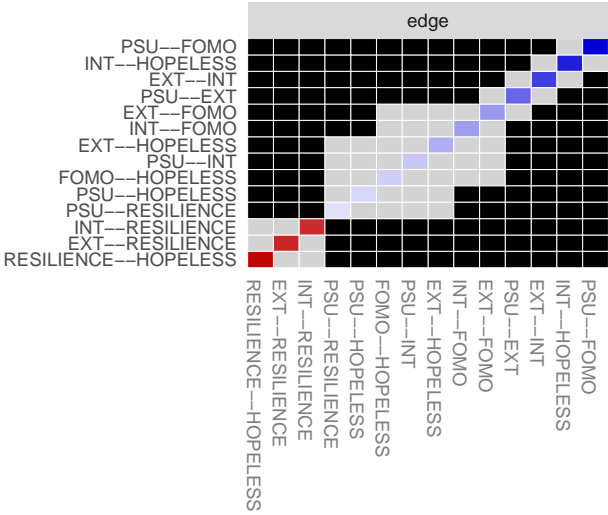

Supplement: Supplementary file 2 — Additional file 2. Supplementary Figure S2: Edge weights differences test results [file 13034_2023_620_MOESM2_ESM.pdf]

Supplementary Figure S3.  
Centrality difference test results

1a (PIU)

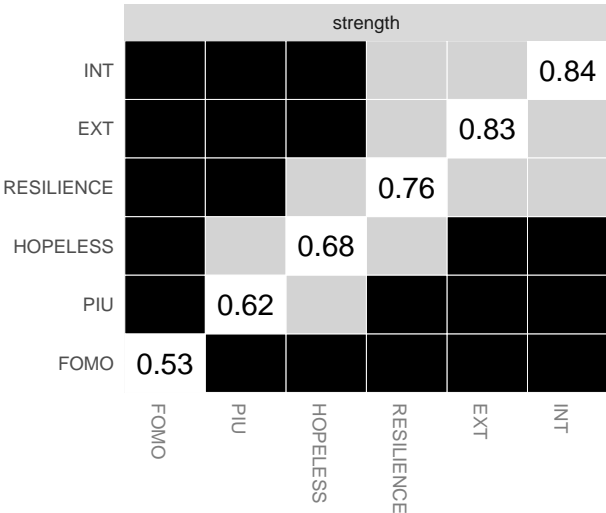

1a (PSU)

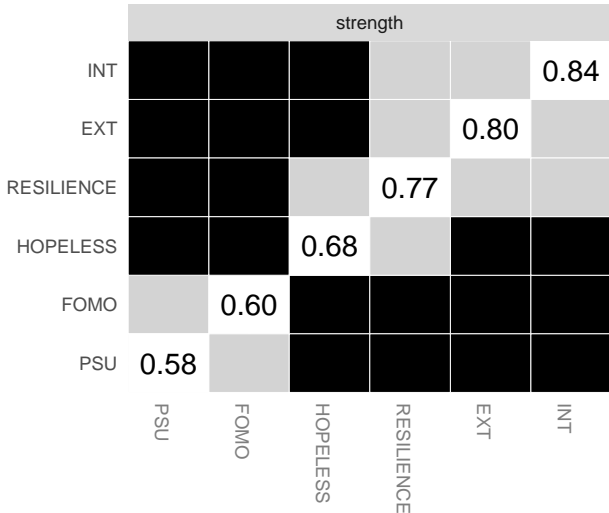

2a (PIU)

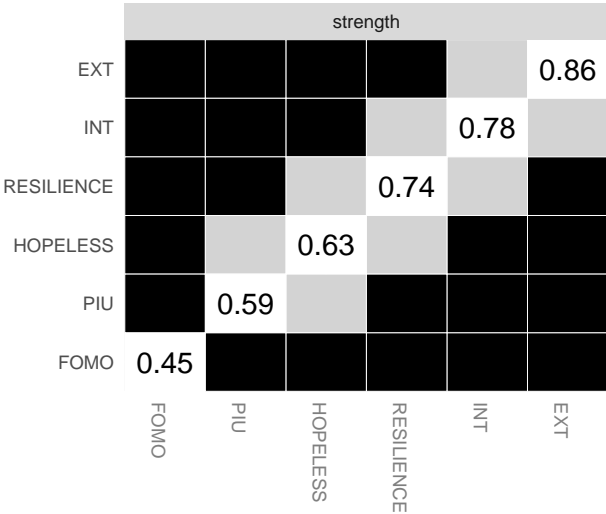

2a (PSU)

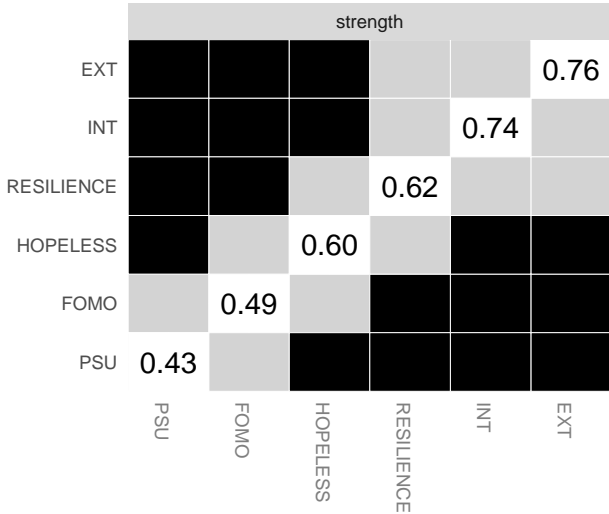

1a (PIU)

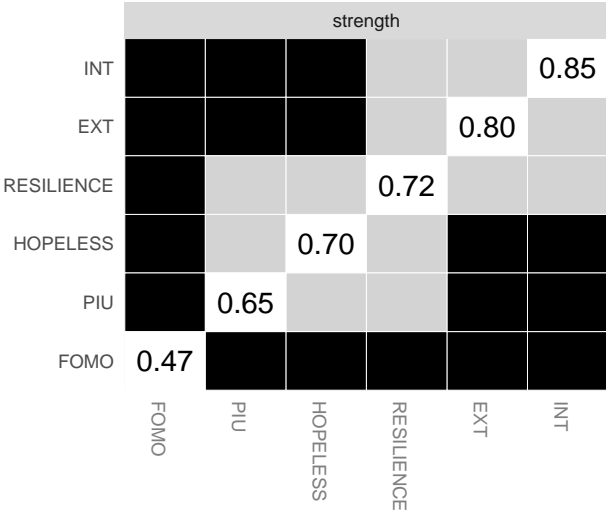

2a (PSU)

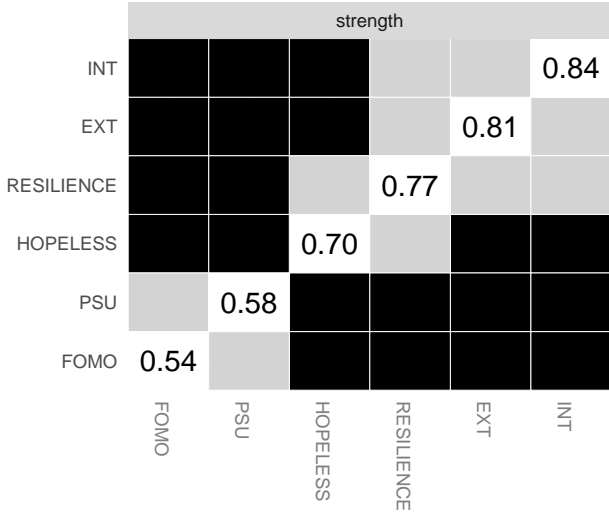

Supplement: Supplementary file 3 — Additional file 3. Supplementary Figure S3: Centrality difference test results [file 13034_2023_620_MOESM3_ESM.pdf]
