# Supplementary material for: Differences between problematic internet and smartphone use and their psychological risk factors in boys and girls: a network analysis
Source: Child Adolesc Psychiatry Ment Health. 2023 Jun 12;17:69. doi: 10.1186/s13034-023-00620-z (PMC10262453; doi:10.1186/s13034-023-00620-z)
Supplement: Supplementary file 4 — Additional file 4. Supplementary Table S1: Correlation analysis results for the total sample, boys, and girls [file 13034_2023_620_MOESM4_ESM.docx]

Supplementary Table 1. *Correlation analysis results for the total sample, boys, and girls*

|  | **Total sample (N = 4070)** | | | | | |  |  |
| --- | --- | --- | --- | --- | --- | --- | --- | --- |
| **Variable** | **1** | **2** | **Paired correlation difference**  **test (PIU-PSU)** | | **3** | **4** | **5** | **6** |
| 1. PIU | - |  |  |  |  |  |  |  |
| 2. PSU | .298*** | - | T | **p** |  |  |  |  |
| 3. Externalizing | .344*** | .230*** | 6.541 | < .001 | - |  |  |  |
| 4. Internalizing | .275*** | .200*** | 4.208 | < .001 | .416*** | - |  |  |
| 5. FoMO | .206*** | .308*** | -5.775 | < .001 | .247*** | .238*** | - |  |
| 6. Resilience | -.244*** | -.076*** | -9.298 | < .001 | -.405*** | -.405*** | -.094*** | - |
| 7. Hopelessness | .262*** | .149*** | 6.292 | < .001 | .321*** | .423*** | .176*** | -.426*** |
|  |  |  | **Boys (N = 2013)** | |  |  |  |  |
| **Variable** | **1** | **2** | **Paired correlation difference**  **test (PIU-PSU)** | | **3** | **4** | **5** | **6** |
| 1. PIU | - |  |  |  |  |  |  |  |
| 2. PSU | .190*** | - | T | **p** |  |  |  |  |
| 3. Externalizing | .328*** | .172*** | 5.800 | < .001 | - |  |  |  |
| 4. Internalizing | .252*** | .148*** | 4.783 | < .001 | .418*** | - |  |  |
| 5. FoMO | .161*** | .270*** | -3.986 | < .001 | .224*** | .180*** | - |  |
| 6. Resilience | -.250*** | -.040 | -7.614 | < .001 | -.384*** | -.379*** | -.073** | - |
| 7. Hopelessness | .248*** | .106*** | 5.148 | < .001 | .295*** | .393*** | .148*** | -.383*** |
|  |  |  | **Girls (N = 2057)** | |  |  |  |  |
| **Variable** | **1** | **2** | **Paired correlation difference**  **test (PIU-PSU)** | | **3** | **4** | **5** | **6** |
| 1. PIU | - |  |  |  |  |  |  |  |
| 2. PSU | .430*** | - | T | **p** |  |  |  |  |
| 3. Externalizing | .361*** | .287*** | 3.386 | .001 | - |  |  |  |
| 4. Internalizing | .306*** | .212*** | 4.191 | < .001 | .417*** | - |  |  |
| 5. FoMO | .261*** | .325*** | -2.885 | .004 | .266*** | .240*** | - |  |
| 6. Resilience | -.240*** | -.115*** | -5.454 | < .001 | -.424*** | -.449*** | -.123*** | - |
| 7. Hopelessness | .277*** | .178*** | 4.368 | < .001 | .340*** | .440*** | .189*** | -.464*** |

Notes. PIU = problematic internet use; PSU = problematic smartphone use; FoMO = fear of missing out. *** p < .001.
